# Tobacco industry pricing strategies for single cigarettes and multistick packs after excise tax increases in Colombia

**DOI:** 10.1136/tobaccocontrol-2022-057333

**Published:** 2022-05-31

**Authors:** Zaineb Danish Sheikh, J Robert Branston, Blanca Amalia Llorente, Norman Maldonado, Anna B Gilmore

**Affiliations:** 1 Tobacco Control Research Group, Department of Health, University of Bath, Bath, UK; 2 School of Management, University of Bath, Bath, UK; 3 Fundación Anáas, Bogotá, Colombia; 4 PROESA - Research Center on Health Economics and Social Protection, Department of Economics, Universidad Icesi, Cali, Colombia

**Keywords:** economics, low/middle income country, price, taxation, tobacco industry

## Abstract

**Introduction:**

Taxes on tobacco products are an efficient way of reducing consumption. However, they are only effective if passed on to consumers with higher prices. This study aims to examine tobacco industry (TI) pricing strategies in response to tax increases, and whether they differ by price segments or presentation (packs or individual sticks) in Colombia. This is the first such academic study in Latin America and the first anywhere to include the market for single sticks.

**Methods:**

Using data on cigarette pricing/taxation from a survey of smokers (2016–2017) and official government data on pricing (2007–2019), the TI’s pricing strategies were examined, split by brand, price segments, different sized packs and single cigarettes.

**Results:**

The TI employed targeted pricing strategies in Colombia: differentially shifting taxes; and launching new brands/brand variants. The industry overshifted taxes when increases were smaller and predictable, but used undershifting more when there was a larger increase in 2017, after which it mostly overshifted on budget and premium (but undershifted mid-priced) brands. The prices for single sticks increased more than the tax increase in 2017 when their consumption also increased.

**Conclusion:**

The pricing strategies identified suggest excise taxes can be increased further, particularly the specific component, to reduce the price gap between brand segments. Brands should be restricted to a single variant along with prohibitions on launching new brands/brand variants. Lastly, since the pricing of single sticks does not match the pattern of packs, more monitoring of their sales and distribution is required, especially since they promote consumption and hinder effective implementation of tobacco tax policies.

**WHAT IS ALREADY KNOWN ON THIS TOPIC**

- Tobacco companies use price-based strategies in high-income countries to minimise the impact of taxation, but it is unclear what they do in low and middle-income countries (LMIC), particularly with single sticks.

**WHAT THIS STUDY ADDS**

- We explore how cigarette prices in Colombia changed between 2007 and 2019, covering both packs and single sticks.
- Taxes were differentially shifted for different price tiers of cigarettes and with the quantity of cigarettes purchased.

**HOW THIS STUDY MIGHT AFFECT RESEARCH, PRACTICE AND/OR POLICY**

- The results suggest more work is needed to understand and address tobacco pricing in LMIC contexts, particularly of single cigarettes.

## Introduction

Higher prices as a consequence of increased tobacco tax are one of the most effective and cost-effective measures available to curb the tobacco epidemic. It has an even greater effect on reducing consumption in low and middle-income countries (LMIC) and among youth.^1–4^ The WHO Framework Convention for Tobacco Control (FCTC) calls for higher taxes and prices for tobacco products in order to reduce tobacco-related morbidity and mortality.^5^ However, it is a relatively underused tobacco control measure,^6 7^ with, as of 2021, only 13% of the world’s population living in the 40 countries that meet the WHO’s recommendation of taxing at 75% or greater of the retail price of the most popular cigarette brands.^8–10^

Colombia is one such country where excise taxes on tobacco products fall short of the 75% benchmark.^9^ It scored 3.38 out of 5 on the 2021 Tobacconomics cigarette tax scorecard which assesses countries’ cigarette tax policies in relation to widely accepted best practices.^11^ This score represents a considerable increase on the 2016 score, 2.38,^12^ but shows there is still room for substantial improvement, particularly in cigarette prices and the tax share of price. Indeed, despite several reforms with limited tax increases between 1997 and 2010,^13^ taxation on tobacco remained one of the lowest in Latin America in 2016, while the smoking prevalence was one of the highest at 32.2%, particularly among children aged 13–15 years.^14–17^ In 2016, the nominal price of 20 Marlboro cigarettes was 3872 Colombian pesos (COP$) (then US$0.97).^18 19^

In 2017, as part of a larger fiscal reform, a major excise tax increase on tobacco products was introduced along with an increase in the general value-added tax (VAT).^20^ The reform doubled the specific component of the excise tax from COP$700 (US$0.23) to COP$1400 (US$0.47) per 20-stick pack (the ad valorem tax remained unchanged at 10% of the retail price charged to the public), while VAT was increased from 16% to 19% of the base price. The specific tax increased to COP$2100 (US$0.74) in 2018, and from 2019, an annual tax escalator was implemented where the specific tax is increased by the country’s annual rate of inflation plus four percentage points each year.^21 22^ These changes in tax were expected to substantially decrease tobacco consumption by increasing the retail price of tobacco. However, retail prices are established by the tobacco industry (TI) who do not have to pass on the increased taxes (as it may not align with their profit-making strategies) and hence can (somewhat) mitigate the impact on consumption. In Colombia, the market is essentially contested between the two transnationals, British American Tobacco (BAT; 55% market share according to Euromonitor) and Philip Morris International (PMI; 43%),^23^ giving each considerable market, and hence pricing power. Previous research highlights that the TI employs a variety of pricing strategies to undermine tax increases in many countries.^24–26^

TI pricing tactics are an area of increasing concern worldwide as they weaken the effectiveness of tobacco tax policies. However, relatively few studies have examined the TI’s price-based responses to taxation, with the majority from high-income countries (HIC)^27–30^ such as the UK^31–34^ and USA,^35–41^ and a smaller number exploring LMICs.^24^ In particular, six broad pricing strategies have been identified that are consistently used across different countries: differential tax shifting (both overshifting and undershifting); launching new brand variants/products; product promotions; smoothing prices after a tax increase (ie, avoiding quit-inducing jump in prices via smaller, incremental more frequent adjustments to prices); disguising price increases by reducing the number of sticks per pack (where legally allowed); and changing product attributes or production processes.^24 31^ These TI approaches have not been prominent in taxation discussions in LMICs, where tobacco taxes are relatively low compared with HICs and cigarette affordability is often increasing as a result of income growth. In addition, one particular aspect of concern regarding industry’s price-based responses to taxation that has been ignored thus far is around the pricing of single cigarette sticks which is a significant issue in LMICs (although not necessarily entirely controlled by the TI). By obviating the need to buy a pack, the sale of single sticks makes tobacco more affordable, hindering effective taxation policies^42^ while also providing distribution channels for illicit cigarettes. It might therefore provide the TI with an additional avenue to react to, and undermine, taxation.^43–45^ Although it is illegal to sell loose cigarettes in Colombia since it adopted the FCTC’s Article 16 in 2009 (which prohibits the sale of loose cigarettes or in small packages), they continue to be sold, especially by a large volume of street vendors. Moreover, there is some anecdotal evidence from Colombia that the TI works closely with retailers, and that they take advantage of and encourage the informal market (where single sticks are sold). Consequently, Colombia represents an interesting market to explore the TI’s price-based responses to tax increases with potentially many lessons to learn, especially since no academic study to date (that we are aware of) has considered a Latin American country.

The aim of this study is therefore to address these gaps in knowledge by exploring tobacco pricing in Colombia, including single sticks. To triangulate our analysis, we use two data sources: a survey of the smokers (2016–2017) and official government data on prices (2007–2019). Given the limited studies on the TI’s tax pass-through in LMICs and the lack of research on pricing of single sticks (despite their growing rate of sales in multiple countries),^46–48^ this study will be relevant globally.

## Methods

### Data sources

The first data source analysed is ‘the Demand for Illicit Cigarettes Survey for Colombia’ (DEICS-COL), which is a nationally representative cross-sectional survey of smokers aged 12–65 years carried out in two waves (2016 and 2017) by Fundación Anáas (an independent civil society organisation promoting public health). The 2016 survey was carried out 4 months before a major tax increase in Colombia while the 2017 wave was collected 8 months after. Details of the study enrolment and protocols have been described previously.^43 49 50^ The interviewer-administered survey involved a mix of two methods: smoker’s self-report on consumption pattern, brand and last purchase information including pack size, price paid and place of purchase; and interviewer’s direct observation of cigarette packs/sticks to validate self-reporting. Each wave contained 1697 respondents.

The second source is national-level average cigarette prices issued twice annually (semester I in December and semester II in June each year) by the National Administrative Department of Statistics (DANE). DANE publishes, for the purposes of identifying the price levels on which the excise duties due, the retail prices for all cigarette brands/brand variants (henceforth brands for simplicity) available in supermarkets,^18^ which represents approximately half of the total market. (The remainder of the market is dominated by the informal sector where retail prices will vary more.) For simplicity and consistency, we analysed the semester II data for each year between 2007 and 2019.

### Analysis

We explored cigarette pricing from both data sources separately to understand whether tax changes were entirely translated into price changes or they were shifted differentially, that is, undershifted or overshifted or both. We also explored changes to the brands that were available/used. Since our data cover retail prices, we are unable to distinguish between the wholesale pricing behaviours of the TI and any impact that retailers/distributors had with their own pricing decisions. Furthermore, for simplicity, we treated all sales of loose sticks as if they were duty-paid sales because a significant part of this market is the resale of legal, duty-paid cigarettes bought in multistick packs, and which would therefore be impacted by the tax change. Moreover, such an impact would likely also affect the selling price of illicit products via such channels. All prices are reported in COP and were adjusted to real prices by removing the impact of inflation using the World Bank’s consumer price index^51^ measure of inflation in Colombia, with 2017 chosen as the base year (as the second year of the survey).

#### DEICS-COL survey

Univariate descriptive analysis was used to characterise trends in cigarette prices, tax changes and, hence, net prices (the industry’s earnings from sales once all taxes have been paid). The analyses were conducted using SPSS V.26. Packs were categorised by their number of cigarettes (10, 14, 18 and 20). Cigarette brands were segmented into three hierarchical price categories (economy, mid-priced and premium) by the authors based on the price points in the market over the whole period of the study, following the WHO approach of weighted-average price tertiles.^52 53^

In order to compare packs of different sizes we used price per stick as the key measure. Where it was not provided in the data, we calculated price per stick by dividing the reported per-pack price by the number of sticks in the pack. The taxes paid per individual cigarette were then calculated for each brand based on its selling price. The net price per stick for each price segment was calculated by subtracting the total tax (excise (specific and ad valorem) plus VAT) from the total price per stick. Tobacco tax pass-through for each segment was calculated by calculating the changes in net price per stick. The percentage change in price attributable to government tax was calculated by dividing the total tax increase by the total price increase and then multiplying by 100, while the percentage change due to TI revenue was calculated by dividing the increase in net revenue by the price increase and then multiplying by 100. We also calculated the frequency of smokers reporting using brands in the different market segments and how these changed between the two survey waves. Such changes were tested for significance using χ^2^ tests.

#### DANE data

The data from DANE contained information on the price of packs of 20 cigarettes between 2007 and 2019 (based on data availability). The tax paid per pack (excise and VAT) was calculated based on the price for each brand. Average net price per pack for each brand was then calculated by subtracting the total tax paid from the average price. This was used to track how taxes were shifted to price. We calculated changes in net price by subtracting the net price of the previous year from the net price of the current year.

## Background results

The DEICS-COL survey has been used before; we present a summary of it here as appropriate background to enable our results to be put into context. For more details, please see the previous publications,^43 49 50^ and the online supplemental appendix for a summary table.

10.1136/tobaccocontrol-2022-057333.supp1 Supplementary data

After the tax increase in 2017, the average self-reported price for cigarettes increased for all price categories (online supplemental table 1) and more smokers purchased cigarettes from street vendors.^43^ As observed in both years, more smokers bought singles at their last purchase as compared with packs and cartons, and the frequency increased from 61.8% in 2016 to 73% in 2017 which was statistically significant.^50^ In regard to the sale of packs, a pack of 10 sticks is more commonly purchased as compared with packs of 20, 18 or 14 (a single observation in 2017). After the tax increase, the frequency of purchasing all varieties of packs also decreased by 11% (significant at 1% confidence level).^43^

## Results

### Comparison of DEICS-COL survey prices with DANE prices

**Figure 1:**
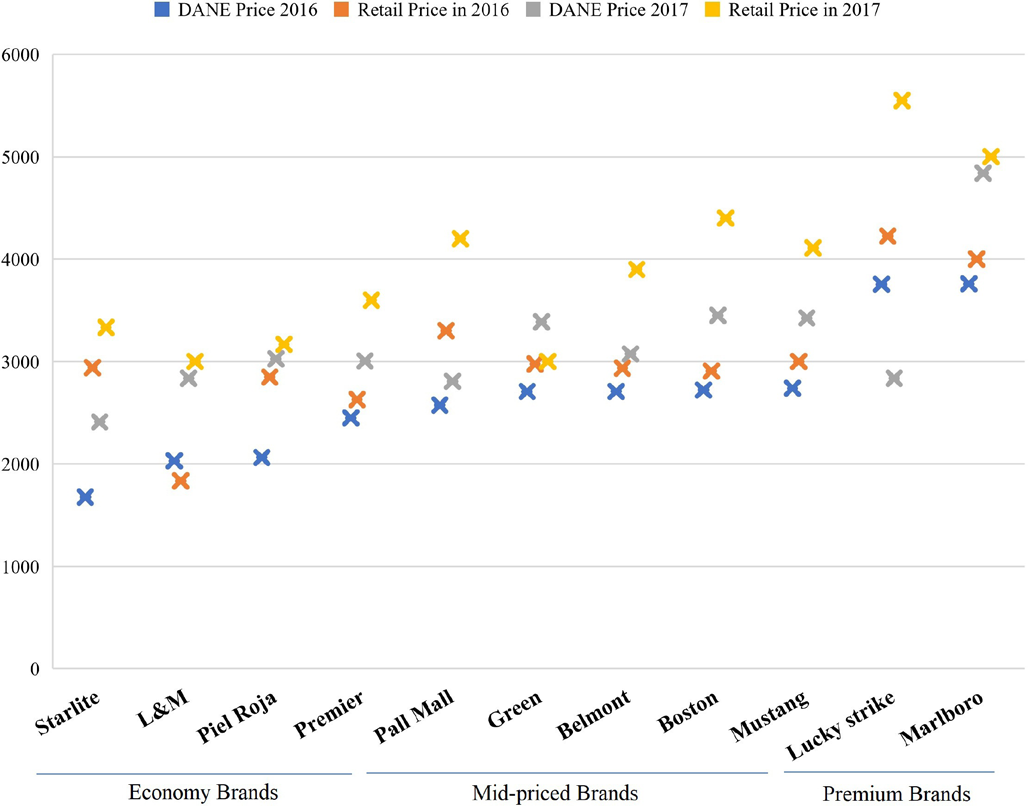
Consistency of Demand for Illicit Cigarettes Survey for Colombia (DEICS-COL) survey retail prices with National Administrative Department of Statistics (DANE) prices for packs of 20 (2016–2017). Source: Authors’ own calculations using the database of DANE and DEICS-COL survey.

A comparison of the survey prices with those from DANE for brands that were present in both the sources revealed that they were broadly consistent. However, the survey prices were generally slightly higher than DANE’s, and these variations were more noticeable in 2017 (figure 1).

### DEICS-COL survey

#### Tax pass-through analysis

An analysis of the changes in the retail price and tax component of survey data between 2016 and 2017 (table 1) showed that for loose sticks the price for all segments increased more than the tax increase, with economy having a greater relative increase in net price followed by mid-priced and premium brands. Similarly, economy packs of 10 and 20 cigarettes were also slightly overshifted. However, taxes on mid-priced and premium packs of 10, 18 and 20 sticks were absorbed to some extent by the industry with their net prices decreasing during the year, signifying undershifting of taxes. These results suggest that the TI has differentially shifted taxes during 2017 for different presentations and pack sizes of cigarettes.

**Table 1:** Changes in the real price and tax for different presentation of cigarettes between 2016 and 2017 (prices per stick; all monetary figures in COP)

Presentation*	Brand categories	Year	Unit price	Price per stick	Specific tax	Ad valorem tax	VAT	Total tax	Net price	Total price increase	Total tax increase	Net price increase	% Price change that is government tax	% Price change that is TI revenue†
Loose sticks‡	Economy	2016	215	215	38	22	30	89	126	85	59	26	70	30
		2017	300	300	70	30	48	148	152					
	Mid-price	2016	323	323	38	32	44	114	208	77	59	18	77	23
		2017	400	400	70	40	64	174	226					
	Premium	2016	430	430	38	43	59	140	290	70	60	10	86	14
		2017	500	500	70	50	80	200	300					
Box of 10	Economy	2016	1075	108	38	11	15	64	44	48	47	1	98	2
		2017	1550	155	70	16	25	111	45					
	Mid-price	2016	1935	194	38	19	27	84	110	37	46	−10	124	−24
		2017	2300	230	70	23	37	130	100					
	Premium	2016	2473	247	38	25	34	97	151	33	47	−14	142	−42
		2017	2800	280	70	28	45	143	137					
Box of 18§	Mid-price	2016	2688	149	38	15	21	74	76	45	46	−2	102	−2
		2017	3500	194	70	19	31	120	74					
Box of 20	Economy	2016	2150	108	38	11	15	64	44	55	49	6	89	11
		2017	3250	163	70	16	26	112	50					
	Mid-price	2016	3440	172	38	17	24	79	93	23	42	−19	183	−83
		2017	3900	195	70	20	31	121	74					
	Premium	2016	4301	215	38	22	30	90	126	40	47	−7	118	−18
		2017	5100	255	70	26	41	137	119					

Source: Authors’ own calculations using the data from the DEICS-COL survey.

*Survey did not find sufficient data on purchase of packs of 14 sticks in 2016, therefore they were removed from the analysis.

†We cannot tell if that is the tobacco industry changing their recommended prices or if that is simply the retailers independently increasing their prices.

‡We treated loose sticks as if they were all subject to taxation.

§Survey only contained data on mid-priced segments of packs of 18 sticks.

COP, Colombian peso; DEICS-COL, Demand for Illicit Cigarettes Survey for Colombia; TI, tobacco industry; VAT, value-added tax.

**Figure 2:**
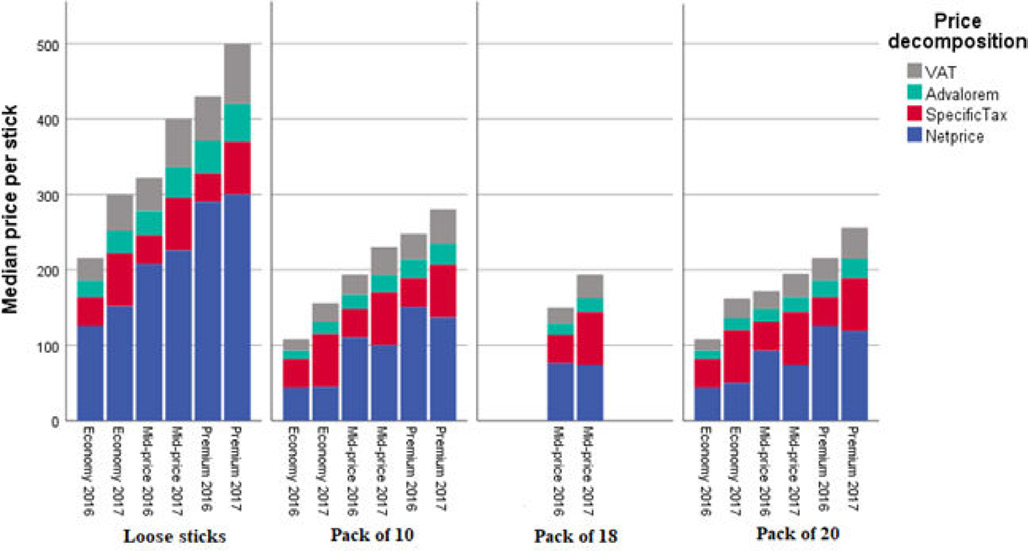
Changes in median price per stick for different presentation of cigarettes, 2016–2017. Source: Authors’ own calculations using the data from the Demand for Illicit Cigarettes Survey for Colombia (DEICS-COL) survey. VAT, value-added tax.

The unit cost of a loose stick is almost double that of a stick purchased in packs of 10, 18 or 20 sticks (figure 2). For cigarettes bought in packs, the highest priced were sold as packs of 10, and the lowest in packs of 18 cigarettes.

An analysis of the popularity of the different market segments (online supplemental table 2) showed that mid-priced cigarettes were most popular in 2016 (51%), but in 2017 it was premium (50%). The frequency of mid-priced brands decreased between the 2 years with a statistically significant shift to economy and premium brands. Additionally, taxes on some brands were more absorbed than others in the same price categories (online supplemental table 3). For example, Marlboro was more undershifted than Lucky Strike (premium), Belmont more than Mustang (mid-price) and D&J more than Starlite (economy). The reasons for this are unclear but it was consistently PMI brands that were more undershifted, so it likely relates to the different profit-maximising strategies of the tobacco companies and the competition that exists between their brands.

### DANE data

#### Descriptive

DANE data analysis revealed that between 2007 and 2019, the industry launched new variants of several existing brands (L&M, Lucky Strike, Marlboro), and this happened throughout the period under analysis, particularly during the most recent years. Furthermore, international brands Chesterfield and Rothmans were introduced as replacements for existing brands (Boston and Mustang, respectively) in 2017–2018 (packs featured both names for a time). Similarly, other new flavour variants with mint/menthol, fruit and/or beverage flavourings were also launched (eg, Lucky Strike Mojito) as were strength variants (eg, L&M Red).

##### Tax pass-through analysis

Examination of the changes in the price and tax components of DANE data revealed that the TI increased prices beyond the tax increase for most brands between 2007 and 2016, although there were a few exceptions in different price categories throughout this period (table 2 and online supplemental table 4). The major tax increase in 2017 brought about a change in the industry pricing strategy from overshifting to undershifting, as seen by the drop in net prices for all brands. However, the price changes between brands that were withdrawn, and new replacement brands/brand variants showed that the prices were notably increased for both Rothmans and Chesterfield beyond that required by the tax increase alone (change in net price between COP$392 and COP$541). Overall, the tax changes in 2018 and 2019 were largely overshifted except for a few mid-priced varieties, including prices for brands that were replaced in the market (Mustang and Boston).

**Table 2:** Year-to-year change in real net price for different brands and brand variants of cigarettes between 2008 and 2019 (adjusted to 2017 COP)

Brand categories	Brands*	Brand variants	2008	2009	2010	2011	2012	2013	2014	2015	2016	2017	2018	2019
Economy	President	Con Filtro	203	90	155	112	24	10	5	−12	28	−287	289	270
	American Gold	Con Filtro	−19	−68	135	−78	89	20	71	24	110	−394	−616	−318
	Starlite	Con Filtro	128	46	224	168	209	−493	-8	9	63	−252	266	351
	Caribe	Caja Blanda	61	70	154	−102	88	20	74	−11	17	−35	172	300
	Caribe	Caja Dura	43	151	225	−189	45	−18	110	−20	65	−257	226	506
	Premier	Azul	161	114	166	110	192	126	137	5	101	−324	314	−388
	Premier	Menthol	152	94	187	184	50	200	140	82	24	−703	−354	−291
	Premier	Rojo	108	135	160	193	36	201	134	11	70	−194	137	−245
	Pielroja	Sin Filtro	−70	−40	240	82	200	40	60	45	104	−116	119	490
Mid-price	Pall Mall	Kristal Frost	122	−35	198	527	−166	156	297	31	75	−506	−607	N/A
	Green	Mentolado	19	117	118	100	189	65	108	104	97	−376	−395	−46
	Derby	Caja Blanda	29	45	334	35	43	54	205	−91	180	−209	−25	547
	Boston	Azul	N/A	N/A	N/A	N/A	N/A	55	110	88	108	−166	−206	516
	Boston	Plata	N/A	N/A	N/A	N/A	N/A	68	82	163	94	−357	−230	−109
	Mustang	Rojo	308	108	55	310	23	64	102	73	127	−412	−441	66
	Mustang	Azul	239	94	97	200	93	52	128	48	166	−200	−404	144
Premium	Rothmans†	Azul	N/A	N/A	N/A	N/A	N/A	N/A	N/A	N/A	N/A	N/A	N/A	506
	Rothmans†	Gris	N/A	N/A	N/A	N/A	N/A	N/A	N/A	N/A	N/A	N/A	N/A	527
	Chesterfield†	Capsula	N/A	N/A	N/A	N/A	N/A	N/A	N/A	N/A	N/A	N/A	88	541
	Chesterfield†	Menthol	N/A	N/A	N/A	N/A	N/A	N/A	N/A	N/A	N/A	N/A	91	392
	Chesterfield†	White	N/A	N/A	N/A	N/A	N/A	N/A	N/A	N/A	N/A	N/A	77	517
	Kent	Blue	−185	−66	172	367	−152	−25	170	-4	33	−720	−610	−147
	Marlboro	Rojo	−294	396	259	203	−46	121	166	62	101	−106	170	761
	Lucky Strike	Red	−36	36	208	210	−517	134	394	16	179	−310	279	878

Brands are categorised into three price categories based on the price points in the market.

Prices adjusted to real prices with 2017 chosen as the base year.

Source: Authors’ own calculations using the database of Colombia’s National Administrative Department of Statistics (DANE).

*Only brands for which pricing information was available for all the years between 2007 and 2019 were included in the analysis.

†These brands were introduced in 2017 and replaced the existing brands.

COP, Colombian peso; N/A, data not available.

## Discussion

This study provides a comprehensive overview of the TI’s pricing strategies in Colombia, including single-stick sales, and therefore contributes to the evidence base on TI’s tax pass-through to consumers in LMICs. Between 2007 and 2016, while tax increases were small, the industry was consistently overshifting taxes, thereby increasing its profitability. This seemed to change following the large tax increase in 2017, when a more complex pattern emerged where taxes on loose cigarettes and economy segments of packs of 10 and 20 were overshifted while they were partially absorbed on packs of mid-priced and premium. That taxes were not shifted equally between packs and single sticks suggests that the pricing of single sticks moves slightly differently from that for packs. From 2017, there is some disagreement between the DANE pricing information and that identified in the survey of smokers as the former showed a substantial amount of undershifting. This difference could be because DANE only records prices of 20-stick packs in the supermarkets, thereby potentially missing out an important part of the market. Given this discrepancy, it would suggest that DANE needs to broaden the scope of their data to capture the informal market, including single-stick prices. Furthermore, since DANE are reporting prices for some brands that have been withdrawn, it seems as if they might also need to improve their technical accuracy and/or their data collection methodologies. After such improvements, the nature of the data might offer future possibilities for further research exploring causal behaviours in this area.

Whenever the TI overshifts taxation it signifies a missed opportunity for government as the higher prices could have been caused by tax increases instead of enhanced industry margins. Since overshifting has continued to be observed in some brands/segments in the years after the large tax increase in 2017, this implies there is further scope for larger tax increases, especially since there was no meaningful increase in illicit sales following the larger increases from 2017.^43^

The tax shifting patterns in Colombia are in concordance with other studies on pricing tactics from other parts of the world, where the industry has been shown to either overshift^29 33 37 54–56^ or undershift^57–62^ taxes, or practise selective overshifting and undershifting on different price segments. For example, in most HICs,^32 34 38 56^ and some LMICs,^63 64^ in response to tax increases, the industry ensures smaller price increases for budget and mid-priced brands while setting relatively higher prices for premium brands. However, in the Colombian cigarette market, taxes on mid-priced (since 2017) and premium (in 2017) brands were largely absorbed which might reflect the different stages of the tobacco epidemic and the particular structure of the markets. Smoking prevalence has reduced in Colombia from 12.9% in 2013 to 8.7% in 2018,^65^ accompanied by a consumption shift towards smoking fewer sticks per day since 2013.^66^ Furthermore, like the survey results herein, Euromonitor data suggest there has been a shift away from mid-priced cigarettes in favour of economy and (to a lesser extent) premium brands.^67^ The pricing of mid-priced brands might therefore be an industry strategy to mitigate these trends away from the middle of the market, and hence a different tactic to demand maximisation that is practised in other LMICs.

The availability of single sticks and their high prevalence among smokers complicates the Colombian market and the TI may be using the informal channels to obscure their pricing tactics, since their prices there are not visible. Furthermore, the existence of such informal channels encourages loose-cigarette sales, thereby weakening the impact of tax increases. From a retailer’s perspective, the profit margins on selling single sticks are substantially more than on packs,^68^ therefore making compliance with the existing law that bans such sales harder to achieve.^48 69^ Indeed, the proportion of smokers who bought singles increased after the 2017 tax increase. Such sales may potentially cause a loss of government revenues as it becomes difficult to assess whether taxes have been paid on single sticks (some will have where legal purchases of multistick packs have been resold as single sticks). These findings indicate the weak governance of the national and subnational enforcement authorities who are in charge of regulating and controlling the distribution of cigarettes in the informal market.^17 48 70^ Considering the structural governance obstacles that perpetuate informal vendors, it is very difficult to eliminate these practices as they require interinstitutional interventions, beyond tobacco control measures. This requires exploring ways to make the tobacco companies accountable for their distribution chain, in combination with interventions directed to retailers that go beyond enforcement.

There was also evidence of the TI using the tactic of introducing new brands/brand variants in the market. This has been observed in other countries such as the UK,^32 34^ Spain,^71^ Bangladesh^58^ and Thailand.^72^ However, unlike these other markets where variants were often cheaper substitutes to appeal to price-sensitive consumers, in Colombia new variants were often flavour/strength related.^34^ Colombia is one of 18 countries across the globe that have a large market share for flavoured cigarettes,^23^ suggested to have exceeded 20% in 2019,^73^ in line with the DEICS-COL survey results that suggest consumers choose brands based more on flavour and less on the price.^49^ The introduction of flavours could be related to efforts to attract and maintain smokers in a declining market, and to offer consumers additional features to justify the increasingly higher prices for cigarettes.

### Strengths and limitations

The limitations of this paper relevant to the survey data are the cross-sectional nature of it, which means determination of causality and hence generalisability is unclear. Furthermore, the timing of the survey waves in 2016 and 2017 means they are now several years old and that we were unable to explore the impact of the tax increase beyond the year immediately after its introduction. The survey data were therefore triangulated with government data on all price segments of cigarettes rendering greater confidence in the broad validity of the results. It should, however, be noted that the DANE data cover the prices on which taxes are to be paid, akin to recommended retail prices, and not the actual prices that were actually charged to consumers, which may differ between retailers. In regard to the evaluation of the tax pass-through, we cannot say for certain if their prices were overshifted by the retailers or the TI, and this is especially true with the informal sector selling single sticks where the industry is likely to have less influence. We also treated all loose sticks as if they were duty-paid sales (even though single-stick sales are not legal) but previous work has reported that in 2016/2017, 3.5% of the tobacco market consists of smuggled cigarettes^49^ so the true impact of the tax increase may not have been fully considered. We also did not evaluate the rate of tax pass-through for other forms of tobacco such as roll-your-own, or next-generation products as the analysed data sets only contained pricing information for cigarettes, although this is unlikely a serious issue as their use is low in Colombia (eg, heated tobacco product sales made up only 1.2% of Colombia’s tobacco market in 2020).^74 75^ Similarly, we did not explore all six strategies previously identified as being used by the TI to respond to tax increases (eg, price smoothing), as our data sources did not allow us to do so, so we cannot conclude whether the strategies not examined are present, or not, in the market.

Despite these limitations, this study has several strengths. It is the first academic study we are aware of from Latin America that assess the impact of taxation on the TI pricing strategies, and also the first to examine changes in the price of single cigarettes following a major excise tax increase. Furthermore, the findings of the study augment the evidence base on TI pricing strategies especially in an under-researched context of an LMIC.

### Policy recommendations

Tobacco taxes are still relatively low in Colombia so these should continue to be increased, particularly the specific component so that cheaper economy brands do not have a tax advantage, and hence price differentials between brands narrow. Restrictions on limiting brands to one variant, prohibiting the introduction of new brands/variants, along with a ban on flavours would also likely be effective strategies to adopt.

## Conclusion

In this study we have explored how the industry responds to tax increases in Colombia and have identified the pricing strategies employed by the TI to undermine tax increases. Although Colombia is an LMIC, it still exhibits a lot of the trends we have observed in HICs in terms of the industry tactics. Moreover, we have explored single-stick sales and found that their pricing does not exactly follow the same pattern as packs, suggesting their sales and pricing need to be monitored more carefully. Indeed, it suggests further analysis of single cigarette pricing and its influence on tobacco control policies in LMICs, and thereby on cessation behaviours, is an essential area for future research.

## Data Availability

The DANE data are freely available from the listed online sources. The DEICS-COL survey data can be shared with researchers who meet the relevant criteria for access—help and information can be obtained directly from Fundación Anáas.
