# Supplementary material for: Tobacco industry pricing strategies for single cigarettes and multistick packs after excise tax increases in Colombia
Source: Tob Control. 2022 May 31;33(1):59–66. doi: 10.1136/tobaccocontrol-2022-057333 (PMC10803973; doi:10.1136/tobaccocontrol-2022-057333)
Supplement: Supplementary data [file tobaccocontrol-2022-057333supp001.pdf]

Appendix Table I: Descriptive results of the Survey

Variables	2016		2017		P-value
	Frequency	Percent	Frequency	Percent	
**Do you smoke Daily?**					**0.12**
No	284	16.7	319	18.8	
Yes	1413	83.3	1378	81.2	
Total	1697	100.0	1697	100.0	
**Place of last purchase**					**0.001**
Bar, restaurant, cafeteria	14	0.8	6	0.4	
Cigar store, liquor store	602	35.5	140	8.2	
Duty Free	2	0.1	2	0.1	
Large departmental stores	99	5.8	63	3.7	
Neighbourhood shop	31	1.8	462	27.2	
San Andresitos	2	0.1	3	0.2	
Service station	5	0.3	3	0.2	
Street vendor	915	53.9	1007	59.3	
Don't remember/Don't know	27	1.6	11	0.6	
Total	1697	100.0	1697	100.0	
**Presentation* of last purchase?**					**0.0005**
Loose sticks	1048	61.8	1239	73.0	
Pack	639	37.7	450	26.5	
Carton	10	0.6	8	0.5	
Total	1697	100.0	1697	100.0	
**Pack size/ Number of cigarettes per pack?**					**0.001**
10	395	23.3	298	17.6	

14	0	0	1	0.1	
18	12	0.7	5	0.3	
20	229	13.5	141	8.3	
Total	636	37.5	450	26.5	
**What did you think of while purchasing your last cigarette?**					**0.028**
Less harmful	78	4.6	49	2.9	
Price	171	10.1	165	9.7	
Taste	1448	85.3	1483	87.4	
Total	1697	100.0	1697	100.0	
➢ *Quantity of cigarettes purchased such as single stick or multi-stick packs or cartons<br>➢ Categorical data compared between groups with the use of Pearson chi-square tests.					

Source: Authors' own calculations, using the data from the DEICS-COL survey

Appendix Table II: Frequency of Smoking with respect to Brands (2016- 2017)

Year	2016	2017	Total
Win	5	9	14
D&J	42	65	107
Gold City	0	1	1
Djarum	1	0	1
Brass	0	3	3
Consul	0	4	4
Empire	0	1	1
Golden Deer	11	7	18
Golden Seal	1	1	2

Fly	3	0	3
Jaisalmer	2	0	2
Modern	3	0	3
American Gold	1	0	1
Ibiza	1	1	2
Piel Roja	37	40	77
Belfort	0	2	2
Starlite	63	57	120
Tropical	1	0	1
Ruby	0	2	2
**Total Economy***	**171**	**193**	**364**
Green	84	95	179
Pall Mall	1	5	6
Premier	10	5	15
Fortuna	4	0	4
Belmont	240	198	438
Meridiano	0	2	2
Motana	0	1	1
Boston	283	128	411
Mustang	232	173	405
Rothmans	0	21	2
L&M	11	17	28
Royal	3	0	3
**Total Mid-priced***	**868**	**645**	**1513**
Jet	14	6	20
Chesterfield	0	169	169

Kool	44	7	51
Camel	1	1	2
Lucky Strike	187	368	555
Marlboro	410	307	717
Montreal	1	1	2
Nat Sherman	1	0	1
**Total Premium***	**658**	**859**	**1517**
**Total**	1697	1697	3394
*Pearson chi-square tests significant at the 1% level (p-value = 0.000)			

Source: Authors' own calculations, using the database of Colombia's National Administrative Department of Statistics (DANE).

Appendix Table III: Changes in the real price and tax for different presentation of cigarette Brands between 2016-2017 (all monetary figures in COP)

Brands	No. of cigarettes	Total price 2016	Total price 2017	Specific Tax 2016	Ad valorem 2016	Excise tax 2016	VAT 2016	Total tax 2016	Net price 2016	Specific Tax 2017	Ad valorem 2017	Excise tax 2017	VAT 2017	Total tax 2017	Net price 2017	Total price increase	Total tax increase	Tobacco industry revenue increase	% of price change that is government tax	% of price change that is tobacco industry revenue
**Win**	Loose	100	200	35	10	45	14	59	41	70	20	90	32	122	78	92	59	34	63	37
**D&J**	Loose	175	200	35	17.5	52.5	24	77	98	70	20	90	32	122	78	12	40	-28	334	-234
**Starlite**	Loose	200	300	35	20	55	28	83	117	70	30	100	48	148	152	85	59	26	70	30
**Piel Roja**	Loose	200	300	35	20	55	28	83	117	70	30	100	48	148	152	85	59	26	70	30
**Premier**	Loose	200	300	35	20	55	28	83	117	70	30	100	48	148	152	85	59	26	70	30
**L&M**	Loose	300	300	35	30	65	41	106	194	70	30	100	48	148	152	-23	34	-56	-149	249
**Mustang**	Loose	300	400	35	30	65	41	106	194	70	40	110	64	174	226	77	59	18	77	23
**Belmont**	Loose	300	400	35	30	65	41	106	194	70	40	110	64	174	226	77	59	18	77	23

**Boston**	Loose	300	400	35	30	65	41	106	194	70	40	110	64	174	226	77	59	18	77	23
**Green**	Loose	300	400	35	30	65	41	106	194	70	40	110	64	174	226	77	59	18	77	23
**Marlboro**	Loose	400	500	35	40	75	55	130	270	70	50	120	80	200	300	70	60	10	86	14
**Kool**	Loose	400	500	35	40	75	55	130	270	70	50	120	80	200	300	70	60	10	86	14
**Lucky Strike**	Loose	500	500	35	50	85	69	154	346	70	50	120	80	200	300	-38	34	-72	-91	191
**Pall Mall**	10	1000	2200	350	100	450	138	588	412	700	220	920	351	1271	929	1125	639	486	57	43
**Starlite**	10	1000	1550	350	100	450	138	588	412	700	155	855	247	1102	448	475	470	4	99	1
**L&M**	10	1200	1800	350	120	470	166	636	564	700	180	880	287	1167	633	510	484	26	95	5
**Premier**	10	1450	2000	350	145	495	200	695	755	700	200	900	319	1219	781	441	472	-31	107	-7
**Mustang**	10	1800	2500	350	180	530	248	778	1022	700	250	950	399	1349	1151	565	512	52	91	9
**Green**	10	1800	2500	350	180	530	248	778	1022	700	250	950	399	1349	1151	565	512	52	91	9
**Belmont**	10	1800	2300	350	180	530	248	778	1022	700	230	930	367	1297	1003	365	460	-96	126	-26
**Boston**	10	1800	2400	350	180	530	248	778	1022	700	240	940	383	1323	1077	465	486	-22	105	-5
**Marlboro**	10	2300	2800	350	230	580	317	897	1403	700	280	980	447	1427	1373	327	462	-135	141	-41
**Lucky Strike**	10	2400	3000	350	240	590	331	921	1479	700	300	1000	479	1479	1521	420	489	-69	116	-16
**Boston**	18	2000	4400	630	200	830	276	1106	894	1260	440	1700	703	2403	1997	2250	1214	1036	54	46
**Piel Roja**	18	2500	3500	630	250	880	345	1225	1275	1260	350	1610	559	2169	1331	812	852	-40	105	-5
**Montreal**	20	1000	4000	700	100	800	138	938	62	1400	400	1800	639	2439	1561	2925	1430	1495	49	51
**Golden Seal**	20	1300	3000	700	130	830	179	1009	291	1400	300	1700	479	2179	821	1602	1094	508	68	32
**Golden Deer**	20	1500	2150	700	150	850	207	1057	443	1400	215	1615	343	1958	192	537	822	-285	153	-53
**D&J**	20	1600	2000	700	160	860	221	1081	519	1400	200	1600	319	1919	81	280	757	-478	271	-171
**Starlite**	20	2000	3250	700	200	900	276	1176	824	1400	325	1725	519	2244	1006	1100	980	120	89	11
**Win**	20	2000	1800	700	200	900	276	1176	824	1400	180	1580	287	1867	-67	-350	603	-953	-172	272
**Piel Roja**	20	2050	3500	700	205	905	283	1188	862	1400	350	1750	559	2309	1191	1296	1032	264	80	20

**L&M**	20	2100	3000	700	210	910	290	1200	900	1400	300	1700	479	2179	821	742	889	-147	120	-20
**Premier**	20	2500	3600	700	250	950	345	1295	1205	1400	360	1760	575	2335	1265	912	943	-31	103	-3
**Green**	20	2900	3000	700	290	990	400	1390	1510	1400	300	1700	479	2179	821	-118	685	-802	-581	681
**Mustang**	20	3200	4800	700	320	1020	441	1461	1739	1400	480	1880	766	2646	2154	1360	1075	284	79	21
**Belmont**	20	3200	3900	700	320	1020	441	1461	1739	1400	390	1790	623	2413	1487	460	842	-382	183	-83
**Pall Mall**	20	3300	4200	700	330	1030	455	1485	1815	1400	420	1820	671	2491	1709	652	894	-242	137	-37
**Jet**	20	3400	8000	700	340	1040	469	1509	1891	1400	800	2200	1277	3477	4523	4345	1855	2490	43	57
**Marlboro**	20	4000	5100	700	400	1100	552	1652	2348	1400	510	1910	814	2724	2376	799	948	-149	119	-19
**Lucky Strike**	20	4200	5550	700	420	1120	579	1699	2501	1400	555	1955	886	2841	2709	1034	1014	20	98	2

Source: Authors' own calculations, using the database of Colombia's National Administrative Department of Statistics (DANE).

Appendix Table IV: Tax pass through for cigarette brands between 2007-2019 (all monetary figures in COP)

**Year**	**Price Decomposition**	**President** Con Filtro	**Caribe** Caja Blanda	**Caribe** Caja Dura	**Premier** Azul	**Premier** Menthol	**Premier** Rojo	**Starlite** Con Filtro	**Pielroja** Sin filtro	**American Gold** Con Filtro	**Derby** Caja Blanda	**Pall Mall** Kristal Frost	**Mustang** Rojo	**Mustang** Azul	**Boston** Azul	**Boston** Plata	**Green** Mentolado	**Kent** Blue	**Marlboro** Rojo	**Lucky Strike** Red
**2007**	Unit price	1407	1652	1671	1849	1884	1946	1960	1975	2399	2637	2294	2241	2323	N/A	N/A	2550	4800	4034	4565
	Total tax	1237	1293	1297	1338	1346	1360	1364	1367	1464	1519	1440	1428	1447	N/A	N/A	1499	2014	1838	1960
	Net price	170	359	373	511	538	586	596	608	935	1118	854	813	877	N/A	N/A	1051	2787	2195	2605
**2008**	Unit price	1616	1664	1658	1984	2005	2007	2047	1802	2275	2566	2360	2553	2541	N/A	N/A	2469	4359	3481	4329
	Total tax	1251	1263	1261	1339	1344	1344	1354	1296	1408	1477	1428	1474	1472	N/A	N/A	1454	1904	1695	1897
	Net price	365	401	397	645	661	662	693	506	867	1089	932	1079	1070	N/A	N/A	1015	2455	1786	2432

	Change in net price	203	61	43	161	152	108	128	-70	-19	29	122	308	239	N/A	N/A	19	-185	-294	-36
**2009**	Unit price	1657	1675	1776	2032	2026	2080	2002	1660	2065	2486	2188	2556	2527	N/A	N/A	2490	4016	3802	4121
	Total tax	1226	1230	1254	1315	1314	1327	1308	1227	1323	1423	1352	1440	1433	N/A	N/A	1424	1787	1736	1812
	Net price	431	445	522	717	713	754	694	433	742	1063	836	1116	1094	N/A	N/A	1066	2229	2066	2309
	Change in net price	90	70	151	114	94	135	46	-40	-68	45	-35	108	94	N/A	N/A	117	-66	396	36
**2010**	Unit price	1713	1729	1919	2087	2110	2126	2135	1827	2079	2744	2279	2445	2472	N/A	N/A	2464	4000	3909	4148
	Total tax	1145	1148	1194	1234	1239	1243	1245	1172	1232	1390	1279	1319	1325	N/A	N/A	1323	1689	1667	1724
	Net price	569	580	725	853	871	883	890	655	847	1354	1000	1126	1147	N/A	N/A	1141	2312	2242	2424
	Change in net price	155	154	225	166	187	160	224	240	135	334	198	55	97	N/A	N/A	118	172	259	208
**2011**	Unit price	1847	1580	1652	2210	2329	2357	2333	1918	1955	2754	2944	2822	2704	N/A	N/A	2565	4417	4113	4355
	Total tax	1179	1115	1132	1265	1293	1300	1294	1196	1204	1394	1440	1411	1383	N/A	N/A	1350	1790	1718	1776
	Net price	668	465	520	945	1035	1057	1038	722	750	1359	1505	1411	1322	N/A	N/A	1216	2627	2395	2580
	Change in net price	112	-102	-189	110	184	193	168	82	-78	35	527	310	200	N/A	N/A	100	367	203	210
**2012**	Unit price	1854	1681	1694	2425	2354	2363	2567	2154	2044	2755	2666	2796	2774	2804	2791	2765	4109	3953	3570
	Total tax	1184	1143	1146	1320	1303	1305	1354	1256	1229	1399	1378	1408	1403	1411	1407	1401	1721	1684	1592
	Net price	670	538	548	1105	1051	1058	1213	898	814	1357	1289	1387	1371	1394	1384	1364	2388	2270	1977
	Change in net price	24	88	45	192	50	36	209	200	89	43	-166	23	93	N/A	N/A	189	-152	-46	-517
**2013**	Unit price	1837	1681	1645	2543	2571	2581	1867	2166	2033	2768	2815	2820	2783	2817	2820	2792	3975	4017	3661

	Total tax	1177	1140	1132	1345	1352	1354	1185	1256	1224	1399	1410	1411	1403	1410	1411	1405	1686	1696	1611
	Net price	659	541	513	1198	1219	1226	683	910	809	1369	1405	1409	1381	1407	1409	1387	2289	2321	2050
	Change in net price	10	20	-18	126	200	201	-493	40	20	54	156	64	52	55	68	65	-25	121	134
**2014**	Unit price	1782	1720	1731	2647	2678	2680	1795	2177	2061	2956	3124	2872	2871	2881	2847	2853	4094	4130	4080
	Total tax	1130	1115	1118	1336	1343	1344	1133	1224	1196	1409	1449	1390	1389	1391	1383	1385	1680	1689	1677
	Net price	652	605	613	1311	1335	1336	661	953	864	1547	1675	1483	1482	1489	1463	1468	2414	2441	2403
	Change in net price	5	74	110	137	140	134	-8	60	71	205	297	102	128	110	82	108	170	166	394
**2015**	Unit price	1791	1733	1732	2655	2786	2694	1832	2250	2110	2829	3153	2963	2929	2990	3056	2985	4049	4171	4063
	Total tax	1170	1156	1156	1376	1407	1385	1180	1279	1246	1417	1494	1449	1441	1455	1471	1454	1707	1736	1710
	Net price	621	577	576	1279	1379	1309	652	971	864	1412	1659	1514	1488	1535	1585	1531	2342	2435	2352
	Change in net price	-12	-11	-20	5	82	11	9	45	24	-91	31	73	48	88	163	104	-4	62	16
**2016**	Unit price	1801	1730	1792	2719	2743	2716	1886	2337	2212	2989	3159	3047	3065	3047	3092	3028	3958	4163	4162
	Total tax	1181	1164	1179	1400	1405	1399	1201	1309	1279	1464	1504	1478	1482	1477	1488	1473	1694	1743	1743
	Net price	620	566	613	1319	1338	1317	685	1029	933	1525	1655	1569	1583	1570	1604	1555	2263	2420	2419
	Change in net price	28	17	65	101	24	70	63	104	110	180	75	127	166	108	94	97	33	101	179
**2017**	Unit price	2282	2554	2314	3111	2622	3283	2410	3026	2531	3525	3286	3306	3610	3639	3424	3336	3762	4788	4512
	Total tax	1993	2063	2001	2208	2081	2252	2026	2186	2057	2315	2253	2258	2337	2345	2289	2266	2377	2643	2572
	Net price	289	491	313	903	541	1031	384	840	474	1210	1033	1048	1273	1294	1135	1070	1385	2145	1940

	Change in net price	-287	-35	-257	-324	-703	-194	-252	-116	-394	-209	-506	-412	-200	-166	-357	-376	-720	-106	-310
**2018**	Unit price	3516	3622	3463	4353	2978	4280	3609	4008	2536	4297	3279	3523	3867	4162	3923	3614	3736	5784	5663
	Total tax	2947	2975	2933	3164	2807	3146	2971	3075	2693	3150	2885	2949	3038	3115	3053	2972	3004	3536	3504
	Net price	569	647	530	1189	170	1135	638	933	-157	1147	393	574	829	1047	870	641	732	2248	2158
	Change in net price	289	172	226	314	-354	137	266	119	-616	-25	-607	-441	-404	-206	-230	-395	-610	170	279
**2019**	Unit price	3992	4132	4256	3917	2706	4040	4190	4766	2239	5125	N/A	3720	4162	4951	3874	3658	3640	6865	6904
	Total tax	3165	3202	3234	3146	2831	3178	3217	3366	2710	3459	N/A	3095	3209	3413	3135	3078	3074	3911	3921
	Net price	827	931	1022	771	-125	862	973	1400	-471	1666	N/A	626	952	1537	740	580	566	2954	2983
	Change in net price	272	300	506	-388	-291	-245	351	490	-318	547	N/A	66	144	516	-109	-46	-147	761	878

*Only brands for which pricing information was available for all the years between 2007-2019 were included in the analysis

**N/A: Data not available

Economy Brands, Mid-Priced Brands, Premium Brands

Source: Authors' own calculations, using the database of Colombia's National Administrative Department of Statistics (DANE).
